# African evolutionary history inferred from whole genome sequence data of 44 indigenous African populations

**DOI:** 10.1186/s13059-019-1679-2

**Published:** 2019-04-26

**Authors:** Shaohua Fan, Derek E. Kelly, Marcia H. Beltrame, Matthew E. B. Hansen, Swapan Mallick, Alessia Ranciaro, Jibril Hirbo, Simon Thompson, William Beggs, Thomas Nyambo, Sabah A. Omar, Dawit Wolde Meskel, Gurja Belay, Alain Froment, Nick Patterson, David Reich, Sarah A. Tishkoff

**Affiliations:** 1 Department of Genetics, University of Pennsylvania, Philadelphia, PA 19104 USA; 2 Present Address: State Key Laboratory of Genetic Engineering, Human Phenome Institute, School of Life Sciences, Fudan University, 2005 Songhu Road, Shanghai, China; 3 Department of Genetics, Harvard Medical School, Boston, MA 02115 USA; 4 Broad Institute of Harvard and MIT, Cambridge, MA 02142 USA; 5 Howard Hughes Medical Institute, Harvard Medical School, Boston, MA 02115 USA; 6 Present Address: Division of Genetic Medicine, Vanderbilt University Medical Center, Vanderbilt University, Nashville, TN 37232 USA; 7 Department of Biochemistry, Muhimbili University of Health and Allied Sciences, Dares Salaam, Tanzania; 8 Center for Biotechnology Research and Development, Kenya Medical Research Institute, Nairobi, Kenya; 9 Department of Biology, Addis Ababa University, Addis Ababa, Ethiopia; 10 UMR 208, IRD-MNHN, Musée de l’Homme, Paris, France; 11 Department of Biology, University of Pennsylvania, Philadelphia, PA 19104 USA

**Keywords:** African populations, Genomic variation, Human evolution, Local adaptation, Demographic history, Effective population size, Whole genome sequencing

## Abstract

**Background:**

Africa is the origin of modern humans within the past 300 thousand years. To infer the complex demographic history of African populations and adaptation to diverse environments, we sequenced the genomes of 92 individuals from 44 indigenous African populations.

**Results:**

Genetic structure analyses indicate that among Africans, genetic ancestry is largely partitioned by geography and language, though we observe mixed ancestry in many individuals, consistent with both short- and long-range migration events followed by admixture. Phylogenetic analysis indicates that the San genetic lineage is basal to all modern human lineages. The San and Niger-Congo, Afroasiatic, and Nilo-Saharan lineages were substantially diverged by 160 kya (thousand years ago). In contrast, the San and Central African rainforest hunter-gatherer (CRHG), Hadza hunter-gatherer, and Sandawe hunter-gatherer lineages were diverged by ~ 120–100 kya. Niger-Congo, Nilo-Saharan, and Afroasiatic lineages diverged more recently by ~ 54–16 kya. Eastern and western CRHG lineages diverged by ~ 50–31 kya, and the western CRHG lineages diverged by ~ 18–12 kya. The San and CRHG populations maintained the largest effective population size compared to other populations prior to 60 kya. Further, we observed signatures of positive selection at genes involved in muscle development, bone synthesis, reproduction, immune function, energy metabolism, and cell signaling, which may contribute to local adaptation of African populations.

**Conclusions:**

We observe high levels of genomic variation between ethnically diverse Africans which is largely correlated with geography and language. Our study indicates ancient population substructure and local adaptation of Africans.

**Electronic supplementary material:**

The online version of this article (10.1186/s13059-019-1679-2) contains supplementary material, which is available to authorized users.

## Introduction

Paleontological and genetic evidence indicates that modern humans originated in Africa within the past 300 thousand years (ky) [1] and spread across the globe within the last 100 ky [2]. Therefore, modern humans have continuously inhabited the African continent longer than any other region [2]. Africans have high levels of genetic, cultural, and linguistic diversity [3] as well as extensive population structure [4]. More than 2000 ethnolinguistic groups have been identified in Africa, consisting of around one third of the world’s languages [4]. Almost all African languages are classified into four major phyla: Afroasiatic, Nilo-Saharan, Niger-Congo, and Khoesan [5]. Afroasiatic languages are mainly spoken by agro-pastoralist and agriculturalist populations in northern and eastern Africa. Nilo-Saharan languages are spoken mainly by pastoralists in central and eastern Africa. The Niger-Congo phylum, with 1436 languages, is the largest language phylum in the world. The Bantu languages, which are a subfamily of the Niger-Congo phylum, are a collection of around 500 closely related languages and are spoken by at least 200 million people due to the migration within the last four thousand years of Bantu-speaking people across eastern and southern sub-Saharan Africa (a.k.a. the Bantu expansion) [4, 6]. The Khoesan language phylum, characterized by click consonants, is the smallest of the four language phyla in Africa. Populations classified as speaking Khoesan languages include hunter-gatherer populations in southern Africa, referred to as “San,” as well as the Hadza and Sandawe who are current and former hunter-gatherer populations, respectively, though their languages are highly divergent and their classification as one language family is contentious [5, 7, 8].

African populations practice a wide variety of subsistence patterns including hunting-gathering, pastoralism, fishing, agriculture, and agro-pastoralism [4, 9, 10]. Due to their large long-term population sizes and deep population divergence times compared to non-Africans, Africans have the highest level of genetic diversity in comparison to any other populations in the world [11]. At least 14 genetically defined ancestral clusters were identified in African populations [4]. Due to extensive migration and admixture events, most Africans are genetically heterogeneous with diverse ancestries [4]. Multiple studies have shown that the population substructure evident in African populations today had already begun to develop before anatomically modern humans migrated out of Africa ~ 50–100 kya (thousand years ago) [12–14]. Studying human evolution in Africa also provides numerous textbook examples of local adaptation [15–18]. For example, lactase persistence (LP), the ability to digest lactose in adulthood, is common in populations practicing a pastoralist subsistence but is rare in hunter-gatherer populations [15, 16].

Because all modern humans originated in Africa, a better understanding of the pattern of genetic variation in African genomes is important not just for understanding African demographic history but also, more generally, for deepening our understanding of the origin of modern humans, the genetic basis of adaptation to different environments, and genetic factors influencing disease susceptibility [2, 10, 19]. High-throughput sequencing technologies have provided valuable resources for studying genetic variation in Africans. For example, the 1000 Genome project has sequenced five indigenous African populations, including Esan, Gambian, Luhya, Mende, and Yoruba (all of which speak Niger-Congo languages and originated from West and Central Africa within the past 4 ky), and confirmed that Africans harbor a greater number of genetic variants, both single nucleotide polymorphisms (SNPs) and structural variants (SVs), compared to populations from other continents [20]. A high coverage sequencing study of the genomes of 15 individuals from three African hunter-gatherer populations, central African rainforest hunter-gatherer (CRHG), and Khoesan-speaking Hadza and Sandawe in east Africa, identified novel genetic diversity and signatures of local adaptation in these populations [21]. The African Genome Variation Project conducted whole genome sequencing at low coverage in seven populations [22]. Nonetheless, these studies only cover a small proportion of the genetic diversity in Africa.

**Fig. 1:**
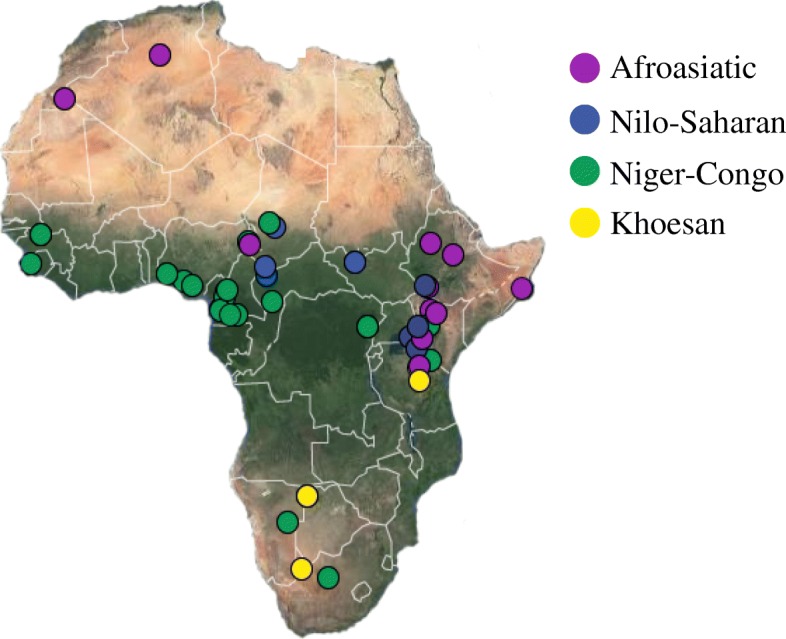
Locations of samples included in this study. Each dot is an individual and the color indicates the language classification

To extend our knowledge of patterns of genomic diversity in Africa, we generated high coverage (> 30×) genome sequencing data from 43 geographically diverse Africans originating from 22 ethnic groups, representing a broad array of ethnic, linguistic, cultural, and geographic diversity (Additional file 1: Table S1). These include a number of populations of anthropological interest that have never previously been characterized for high-coverage genome sequence diversity such as Afroasiatic-speaking El Molo fishermen and Nilo-Saharan-speaking Ogiek hunter-gatherers (Kenya); Afroasiatic-speaking Aari, Agaw, and Amhara agro-pastoralists (Ethiopia); Niger-Congo-speaking Fulani pastoralists (Cameroon); Nilo-Saharan-speaking Kaba (Central African Republic, CAR); and Laka and Bulala (Chad) among others (Additional file 1: Table S1). We integrated this data with 49 whole genome sequences generated as part of the Simons Genome Diversity Project (SGDP) [14] (Fig. 1). Our new dataset, consisting of 92 individuals from 44 indigenous African populations speaking languages belonging to the four main language phyla and practicing diverse subsistence patterns, greatly expands representation of whole genome sequences from geographically, culturally, and linguistically diverse Africans. We constructed phylogenetic relationships and inferred the population structure, effective population size, and divergence time of these populations. In addition, we identified signatures of positive selection in populations that have adapted to diversified environments and diets.

## Results and discussion

We analyzed high-coverage whole genome sequencing data from 92 individuals from 44 indigenous African populations and a comparative dataset consisting of 62 west Eurasian individuals from 32 populations represented in the SGDP [14]. We identified 26,230,650 SNPs and selected a set of 7,497,970 SNPs, after pruning based on linkage disequilibrium (LD), for use in further analyses.

### Phylogenetic relationship of African populations

**Fig. 2:**
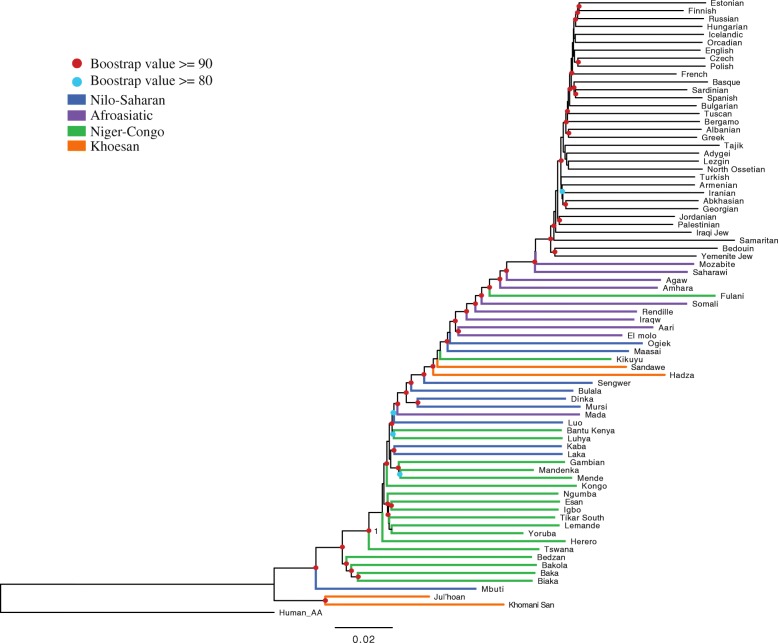
Phylogenetic relationship of 44 African and 32 west Eurasian populations determined by a neighbor joining analysis assuming no admixture. Here, the dots of each node represent bootstrap values and the color of each branch indicates language usage of each population. Human_AA human ancestral alleles

A set of 4,587,274 SNPs for which we could make a high confidence determination of an ancestral allele were used to construct the phylogenetic relationship of Africans and Eurasians using a neighbor-joining (NJ) method, which assumes no admixture events. Thus, individuals who cluster near each other in the tree could either share a recent common ancestry and/or experienced gene flow. The resolution of the reconstruction is excellent; bootstrap values of most nodes are greater than 90. The San lineage (including Khomani San and Juǀ’hoan) is the basal lineage of all modern human lineages. The other African populations mainly cluster in the tree based on their current geographic location, with the exception of the CRHG and some pastoralist/agro-pastoralist populations such as the Mada and Luo, the latter of which have migrated over long distances and admixed with neighboring populations. We found that the CRHG populations from central Africa, including the Mbuti from the Demographic Republic of Congo (DRC), Biaka from the CAR, and Baka, Bakola, and Bedzan from Cameroon, also form a basal lineage in the phylogeny. The other two hunter-gatherer populations, Hadza and Sandawe, living in Tanzania, group with populations from eastern Africa (Fig. 2). The two Nilo-Saharan-speaking populations, the Mursi from southern Ethiopia and the Dinka from southern Sudan, group into a single cluster, which is consistent with archeological data indicating that the migration of Nilo-Saharan populations to eastern Africa originated from a source population in southern Sudan in the last 3000 years [4, 23–25]. The Fulani people are traditionally nomadic pastoralists living across a broad geographic range spanning Sudan, the Sahel, Central, and Western Africa. The Fulani in our study, sampled from Cameroon, clustered with the Afroasiatic-speaking populations in East Africa in the phylogenetic analysis, indicating a potential language replacement from Afroasiatic to Niger-Congo in this population (Fig. 2). Prior studies suggest a complex history of the Fulani; analyses of Y chromosome variation suggest a shared ancestry with Nilo-Saharan and Afroasiatic populations [24], whereas mtDNA indicates a West African origin [26]. An analysis based on autosomal markers found traces of West Eurasian-related ancestry in this population [4], which suggests a North African or East African origin (as North and East Africans also have such ancestry likely related to expansions of farmers and herders from the Near East) and is consistent with the presence at moderate frequency of the −13,910T variant associated with lactose tolerance in European populations [15, 16]. Phylogenetic reconstruction of the relationship of African individuals under a model allowing for migration using TREEMIX [27] largely recapitulates the NJ phylogeny with the exception of the Fulani who cluster near neighboring Niger-Congo-speaking populations with whom they have admixed (Additional file 2: Figure S1). Interestingly, TREEMIX analysis indicates evidence for gene flow between the Hadza and the ancestors of the Ju|‘hoan and Khomani San, supporting genetic, linguistic, and archeological evidence that Khoesan-speaking populations may have originated in Eastern Africa [28–30].

### Population structure in African populations

**Fig. 3:**
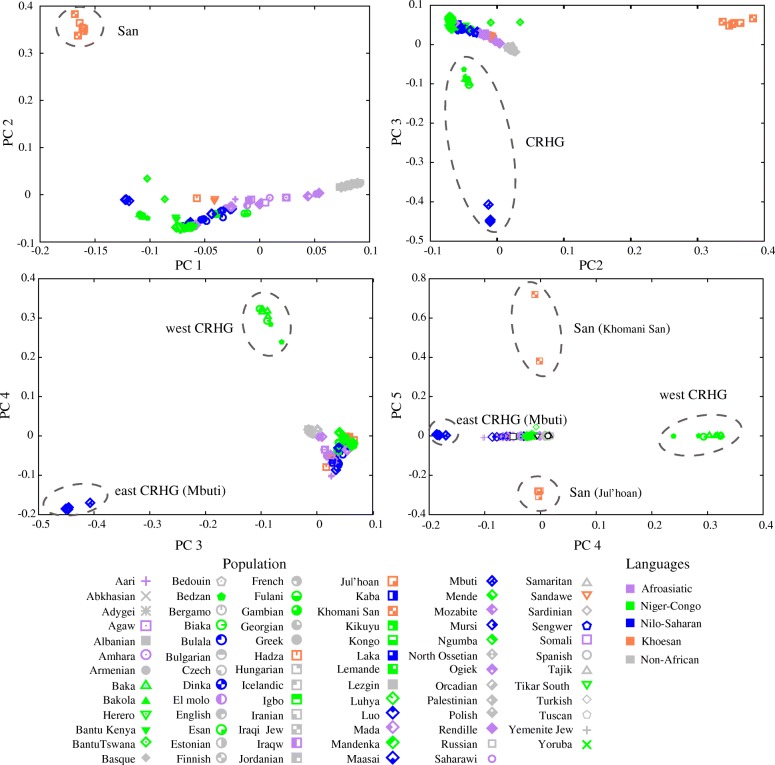
Principal component analysis of 44 African and 32 west Eurasian populations using principal component analysis. Each dot represents an individual and color of dots represents language usage. PC1 separates the African and western Eurasian populations, with Middle-Eastern populations in between. PC2 distinguishes the San populations (both Khomani San and Juǀ’hoan) from other Africans. CRHG individuals (including both eastern and western CRHG) separate with other Africans at PC3

Based on PCA analysis, we found 12 significant principal components (*P* value < 0.05, Tracy–Widom distribution) [31] (Additional file 2: Figure S2). The first PC separates the African and non-African populations, with populations from the Middle East clustering in between. The second PC distinguishes the San populations (both Khomani San and Ju|’hoan) from the rest of the populations. PC3 separates CRHG individuals (including both eastern and western CRHG) from other Africans and PC4 distinguishes eastern and western CRHG individuals (Fig. 3).

**Fig. 4:**
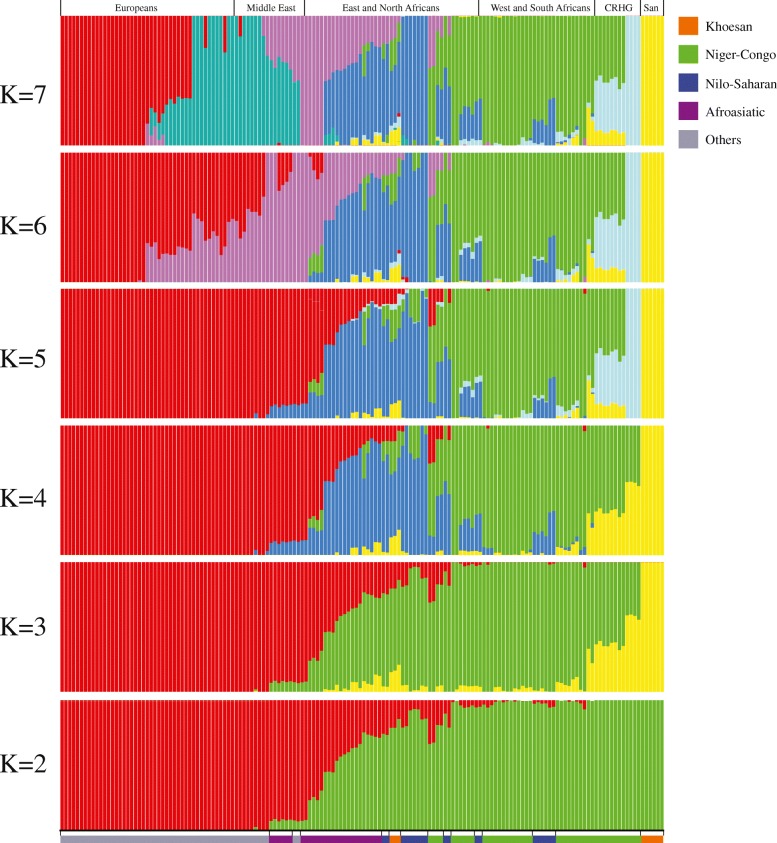
ADMIXTURE analysis of 92 African and 62 West Eurasian individuals. Each bar is an individual and colors represent the proportion of inferred ancestry from *K* ancestral populations. The bottom bar shows the language classification of each individual. *K* = 2 separates the African and West Eurasian populations. African hunter-gatherer populations Khomani San, Ju|’hoan, Sandawe, Hadza, and CRHG populations are distinguished from the rest of the populations at *K* = 3 (yellow bar). From *K* = 5, CRHG populations emerge as a single cluster. With the increasing of *K*, the populations are largely grouped by their current language usage

The ADMIXTURE analysis at *K* = 2 separates the African and West Eurasian populations (Fig. 4, Additional file 2: Figure S3). However, a substantial proportion of West Eurasian-related ancestry was observed in populations located in northern Africa, reflecting historical gene flow among populations in these regions [4, 32]. African hunter-gatherer populations (Khomani San, Ju|’hoan, Sandawe, Hadza, and CRHG) are distinguished from the rest of the populations at *K* = 3. This observation is consistent with previous studies based on autosomal, mitochondrial, and Y-chromosomal markers indicating evidence of ancient-shared ancestry [4, 12, 13, 33]. From *K* = 5, CRHG populations emerge as a single cluster (Fig. 4). With increasing *K* values, the populations are largely grouped by their current language usage but with the same exceptions as described above for the phylogenetic analysis. We find that Bantu-associated ancestry (green bars) is widely spread across populations in eastern and southern Africa. This observation is consistent with archeological and linguistic evidence indicating an expansion of Niger-Congo Bantu-speaking people, which may have originated in the Cross River Valley, a region between South East Nigeria and Western Cameroon, and then dispersed to equatorial, eastern, and southern Africa within the past 3–5 ky [34–36]. Consistent with a proposed Bantu migration, we observe that Niger-Congo ancestry is at the greatest level in western and central African populations (such as Tikar and Lemande) and decreases in eastern (such as Bantu Kenya, Luo, Luhya) and southern (such as Bantu Tswana) African populations. Our ADMIXTURE analyses also suggest that the Sahel-Sudan belt has been a corridor of bidirectional migrations, consistent with [25]. The Sudanese Dinka population has the highest Nilo-Saharan-associated ancestry (blue bars, *K* = 7), which decreases in the East African populations (such as Massai and Luo) and the Western African populations (such as Kaba, Luka, and Bulala), consistent with migration from Sudan westward ~ 7 kya [37] and eastward into Ethiopia, Kenya, and Tanzania within the past 3 ky [4]. Eastern African populations, such as the Luo, Kikuyu, and Bantu from Kenya, show the highest level of admixture in Africa, which reflects the successive migration and admixture events of Bantu, Nilo-Saharan, and Afroasiatic populations into this region within the past 5 ky [4, 35].

### Effective population size (*Ne*) and divergence times

**Fig. 5:**
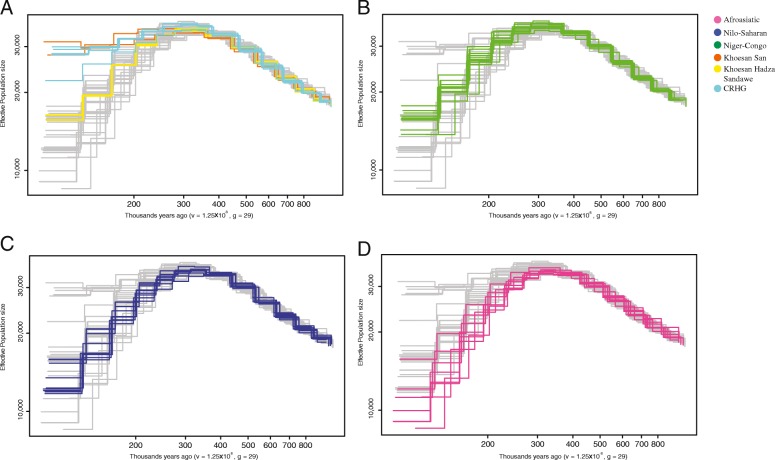
Effective population size inferred using MSMC. Each line represents the average effective population size per population, and the colors represent language usage, except for the CRHG populations. Here, we assume a mutation rate per generate (v) 1.25 × 10^−8^ and average generation time (g) 29 years. **a**–**d** The effective population size of Khoesan-, Niger-Congo-, Nilo-Saharan-, and Afroasiatic-speaking populations

Using the multiple sequentially Markovian coalescent (MSMC) method [38], we found that the *Ne* of Africans started to diverge around 200 kya (Fig. 5), which is consistent with a model of an early emergence of population structure in Africa after the origin of modern humans [4, 39]. Between ~ 200 and ~ 60 kya, the ancestors of Africans who today speak languages belonging to the four major language phyla experienced a common population bottleneck, but not all the populations were affected equally (Fig. 5). The San (including both Khomani San and Juǀ’hoan) maintained the largest *Ne* in this period compared to other populations (Fig. 5a), consistent with prior studies [13, 40]. In addition, we infer that the CRHG populations (including Biaka, Baka, Mbuti, Bedzan, and Bakola, the cyan lines in Fig. 5a), maintained a relatively large *Ne*, which is consistent with higher level of genetic diversity in these populations in comparison to other Sub-Saharan populations [13, 21, 41, 42]. Compared to the San and CRHG populations, the inferred ancestral *Ne* of the Hadza and Sandawe (Fig. 5a, Additional file 2: Figure S4), Niger-Congo-speaking (Fig. 5b) and Nilo-Saharan-speaking populations were lower in this period (Fig. 5c). Afroasiatic-speaking populations (Fig. 5d) in north Africa have the lowest *Ne*, which is also reflected in the elevated LD and the reduction of haplotype diversity in these populations compared to other Sub-Saharan African populations [13, 43, 44]. The low *Ne* in Afroasiatic-speaking populations likely reflect the recent migration and admixture with non-African and north African populations (Fig. 4), whose *Ne* is much lower than Sub-Saharan Africans [39, 45].

**Fig. 6:**
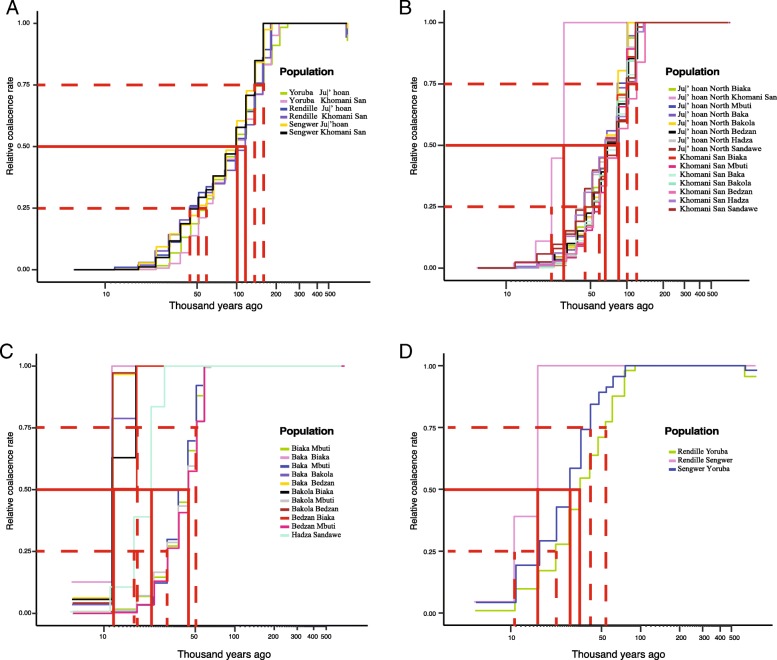
Relative cross-coalescence rate (RCCR) in African populations. Between the San and non-Khoesan-speaking populations (a); between the San and other African hunter-gatherer populations (b); between the CRHG populations and between the Hadza and Sandawe populations (c); between the Nilo-Saharan-, Niger-Congo-, and Afroasiatic-speaking populations (d)

Consistent with the *Ne* analysis, an early emergence of population structure in Africa is supported by the relative cross coalescence rate (RCCR) analysis in MSMC. RCCR models the genetic separation between populations by the ratio of within- and cross-population coalescence rates [38]. For example, an RCCR equal to 50% indicates half of the lineages between a pair of populations descend from a common ancestor. If we consider the time at which 50% of the lineages coalesce (75–25% in parentheses) [14], we estimate that the ancestors of the San and the ancestors of the Niger-Congo, Nilo-Saharan, and Afroasiatic populations were substantially diverged by ~ 120–100 (160–44) kya (Fig. 6a). This estimation agrees with results of previous TMRCA analyses based on mtDNA [33], Y chromosome [46], autosomal microsatellites [47], large-scale SNP genotype data [42], and whole genome sequences [14, 39]. A recent study based on an ancient unmixed San sample at ~ 2 kya suggests more ancient splits between San and other African populations (350 to 260 kya) [48]. If we consider the earliest evidence of population divergence (when RCCR becomes less than one), we observe divergence of the ancestors of current San hunter-gatherers (including both Khomani San and Ju|’hoan) and the ancestors of Niger-Congo-, Nilo-Saharan-, and Afroasiatic-speaking populations at ~ 200 kya (Fig. 6a). In comparison, the inferred divergence time between the San and other African hunter-gatherer populations, such as the CRHG, Hadza, and Sandawe, was inferred to be more recent, though still ancient at 85–68 (120–44) kya (Table 1). The divergence between the ancestors of Juǀ’hoan and Khomani San occurred at ~ 30 (30–24) kya, consistent with prior estimations based on genomic analyses of San populations [49, 50]. Our estimation of times of divergence between eastern and western CRHG at ~ 44 (51–31) kya and between the western CRHG populations at ~ 12 (18–12) kya are comparable to previous estimates [41, 42, 51, 52]. In addition, similar to the estimates based on the Y chromosome and mtDNA variation [12], the two east African Khoesan-speaking populations, the Hadza and Sandawe, diverged ~ 23 (23–17) kya (Table 1) [12, 28]. Although currently, these African hunter-gatherer populations are geographically isolated, analyses based on mitochondrial, Y chromosomal, and autosomal marks suggest these populations could be the remnants of a historically widespread population of hunter-gatherers [4, 12]. For example, a mitochondrial haplotype (L0d), which was mainly observed in populations with San ancestry, was also found in the East African click-speaking Sandawe population who were, until recently, practicing hunting and gathering [53, 54]. In addition, Y chromosome haplotype B2b2 and B2b1-B2b4a lineages were only found in eastern CRHG and south Africa Khoesan-speaking populations [55, 56]. The inferred divergence times between Niger-Congo, Nilo-Saharan, and Afroasiatic-speaking populations suggest that the ancestors of populations speaking these languages shared a common ancestor > 34 kya. Our results suggest that the ancestor of Niger-Congo-speaking populations first split with the ancestor of Nilo-Saharan and Afroasiatic speakers and that the ancestors of Nilo-Saharan and Afroasiatic-speaking populations diverged more recently at ~ 16 kya (16–11 kya) (Table 1). Although the divergence time estimates in this study are largely consistent with previous archeological and genetic studies, future studies that include high coverage whole genome sequencing from a larger number of individuals per population will be particularly informative for applying more complex models of demographic history based on the allele frequency spectrum [57].

**Table 1:** Divergence time estimation between African populations speaking languages belonging to the main language phyla. All the estimates were inferred with MSMC using one individual from each population. CRHG represents central African rainforest hunter-gatherers, including east central African rainforest hunter-gatherers (East CRHG) Mbuti, west central African rainforest hunter-gatherers (West CRHG) Baka, Biaka, Bakola, and Bedzan. San: Khomani San and Ju|'hoan; Niger-Congo: Yoruba. Nilo-Saharan: Sengwer. Afroasiatic: Rendille. CRHG: Baka, Biaka, Bakola, Bedzan, Mbuti. The divergence times that we report here are based on relative cross-coalescent rates at 50% (25–75%)

Population 1	Population 2	Divergence time (kya)
San	Niger-Congo	~ 100 (59–160)
San	Nilo-Saharan	~ 100–120 (44–160)
San	Afroasiatic	~ 100–120 (52–160)
San	Hadza and Sandawe	~ 68–85 (44–100)
San	CRHG	~ 78–85 (52–120)
West CRHG	East CRHG	~ 44 (31–50)
Niger-Congo	Afroasiatic	~ 34 (22–54)
Niger-Congo	Nilo-Saharan	~ 28 (17–41)
Nilo-Saharan	Afroasiatic	~ 16 (11–16)
Hadza	Sandawe	~ 23 (17–23)
Khomani San	Ju|’hoan	~ 30 (24–30)

### Identifying signatures of local adaptation

To identify possible genomic regions contributing to local adaptation among populations, 52 individuals were merged into six “meta-ancestry” groups on the basis of shared ancestry according to *ADMIXTURE* analyses (Fig. 4). This included a CRHG group, consisting of Biaka, Baka, Bakola, Bedzan, and Mbuti individuals; a San group consisting of Ju|‘hoan and Khomani San individuals; a Niger-Congo group consisting of Mandenka, Mende, Yoruba, Igbo, Kongo, and Esan individuals; a Sahel group consisting of Kaba, Laka, and Mada individuals; a Nilo-Saharan group consisting of Sengwer, Dinka, and Mursi individuals; and an Afroasiatic group consisting of Agaw, Amhara, Rendille, and Iraqw individuals. The *d* statistic [58], a sum of normalized, pairwise *F*_ST_ between a focal group and all other populations, was computed for all SNPs with no more than 20% missing data in any group. To identify candidate regions of local adaptation, variants in the top 0.1% of the empirical distribution were considered outliers. To prevent double counting variants in strong LD, all variants with *r*^2^ > 0.5 were grouped together, tagging each group by the variant with the highest *d* value. Variants within 1 Mb and *r*^2^ > 0.8 with the tagging variants were used to define the final selection windows.

We first tested whether locally adaptive variants are enriched in functionally annotated genomic regions, including predicted promoter, dyadic, and enhancer regions from the Roadmap Epigenomics consortium [59], FANTOM5 enhancers [60], GENCODE genic regions [61], and regions conserved across mammals [62]. Using a permutation approach to measure overlap between all outlier variants and functional categories [63], we find that the outlier variants are significantly enriched in predicted promoters, dyadic regions, and enhancers, as well as introns and conserved regions (*P* < 1.0 × 10^−4^ for all tests). Conversely, we find a lack of significant enrichment in FANTOM5 enhancer regions (*P =* 0.23), exons (*P* = 0.97), and 3′ UTRs (*P =* 0.95), highlighting the importance of the noncoding and regulatory genome in the study of human complex and adaptive traits [64].

To detect associations between outlier windows and biological function, we use the Genomic Regions Enrichment of Annotations Tool (GREAT), which tests for gene ontology enrichment of nearby genes [65]. We find genes related to immune function are enriched near outlier windows across several populations (Additional file 3: Table S2), including antimicrobial humoral response in the CRHG (Binomial test, Benjamini-Hochberg *Q* value = 2.2 × 10^−3^), B cell homeostasis in the Niger-Congo and San (*Q* = 4.5 × 10^−3^ and 4.3 × 10^−2^), regulation of phagocytosis and chemokine signaling in the Niger-Congo (*Q* = 1.4 × 10^−2^ and 1.6 × 10^−2^), and cytokine production in the Nilo-Saharan populations (*Q* = 6.2 × 10^−3^). We also see enrichments related to cardiovascular and lipid traits, including response to low-density lipoprotein among the pastoralist Nilo-Saharan and agricultural Niger-Congo populations (*Q* = 1.7 × 10^−4^ and 2.7 × 10^−5^) and regulation of cardiac muscle tissue growth in the Afroasiatic group (*Q* = 4.2 × 10^−7^). Among the San, we find enrichments for loci near genes that play a role in bone morphogenesis (*Q* = 3.0 × 10^−2^), notable due to the relatively gracile bone structure in the San, and near genes that play a role in renal and pancreatic development (*Q* = 6.9 × 10^−3^ and 4.5 × 10^−3^), possible adaptations to low water availability and diet. Outlier windows among the CRHG are also enriched near genes related to abnormal thyrotroph morphology in mice (*Q* = 2.6 × 10^−5^), recapitulating a previously proposed connection between pituitary and thyroid function and the short stature of CRHG [21]. Genes near highly differentiated loci include the transcription factor *POU1F1*, which plays an important role in anterior pituitary development and has been previously identified as a target of selection in these populations [21], as well as *PITX1*, a binding partner of *POU1F1* [66], and the thyroid hormone receptor *THRB* (mutations at this locus can lead to thyroid hormone resistance and goiter [67], which has a relatively low prevalence in CRHG populations [17]). In addition to genes related to pituitary function, genes belonging to a number of growth factor pathways are enriched near CRHG windows, including the fibroblast growth factors *FGF7* and *FGF10*; the fibroblast growth factor receptor *FGFR2*; the bone morphogenetic proteins *BMP2*, *BMP4*, *BMP5*, and *BMP6*; the insulin-like growth factor receptor *IGF2R*; and the insulin-like growth factor binding protein *IGFBP3*. These findings highlight the diversity of genetic and phenotypic variation in Africa and suggest candidate loci underlying several adaptive human phenotypes, such as the short stature of CRHG, as well as possible adaptations to variable environmental pressures such as pathogen burden and diet.

## Conclusion

Anatomically, modern humans originated in Africa within the past 300 kya and have continuously inhabited Africa. Prior studies found that Africans have the highest level of linguistic and genetic diversity compared to the populations in any other continent [4]. Leveraging the whole genome sequences of 92 individuals from 44 African populations, we inferred that the ancestors of present-day populations began to develop substructure as early as ~ 200 kya. Our analyses also identified signatures of multiple waves of migration in Africa, such as the expansion of Bantu-speaking agriculturists from west Africa to eastern and southern Africa, and migration of Nilo-Saharan- and Afroasiatic-speaking populations into East Africa. As these populations migrated and adopted new subsistence strategies, they also encountered novel environments and selective pressures, resulting in local adaptation. Although a low-resolution study suggested limited recent positive selection in Sub-Saharan Africans [68], we found strong signals of positive selection due to local adaptation in the six meta-populations based on regions of high population-specific genomic differentiation, which we find near genes playing important roles in immunity, cardiovascular function, and metabolism. In addition, we find an enrichment of genes related to fibroblast and bone growth factors, as well as pituitary function, among the CRHG populations, providing candidate genes that may underlie their unique short-stature phenotype. An increasing number of publications have identified archaic introgression in modern Africans [48, 69, 70]; its impact on the estimation of population divergence times and effective population sizes needs to be explored. In the future, a combination of phenotypic (such as anthropometric, life history, and metabolic data) and genomic (from both contemporary and ancient samples) data from Africans is needed to better understand the origin and evolution of modern humans, the genetic basis local adaptation, and the evolution of complex traits and related diseases.

## Methods

### Sequencing and SNP calling

The data used in this study are part of the Simons Genome diversity project [14]. They consist of 29 previously published African genome sequences and a novel set of 43 genome sequences from geographically and ethnically diverse Africans from 22 indigenous groups. The full details of the data generation were previously reported [14]. Briefly, all the samples were processed using the PCR-free paired-end library preparation protocol from Illumina. The average insert size is 314 ± 20 bases for libraries. The libraries were sequenced 100 base pairs at each end with average 43-fold coverage using the HiSeq2000 sequencing platform. After trimming the adaptors, the raw reads were aligned to the human reference genome (version hs37d5) using the BWA-MEM version 0.7.12 [71]. The BAM files were stored in the European Nucleotide Archive (accession number PRJEB9586 and ERP010710) and European Genome-phenome Archive (accession number EGAS00001001959). The SNPs were genotyped using the UnifiedGenotyper module in the genome analysis toolkit (GATK) [72], with a modified Wright-Fisher allele frequency spectrum prior to minimize the reference-bias in SNP calling (see more information in the SGDP manuscript). The SNP calling results were stored in the VCF format and hetfa format [14]. We extracted the autosomal SNPs that passed filter level 1 using cTools (https://github.com/mengyao/cTools). To minimize the impact of missing data, we filtered the SNPs in LD using Plink version 1.9 [73] with the parameters --indep-pairwise 50 10 0.1.

### Principal component and ADMIXTURE analyses

We conducted principal component analysis using the smartpca script in the EIGENSOFT toolkit version 6.0.1 [31, 74]. Population structure was inferred using ADMIXTURE version 1.3.0 [75] with randomly starting seeds, 5-fold cross-validation (--cv 5 option) and 100 bootstraps (−B 100). We set the ancestral population number between 2 and 7 (*K* = 2 to 7).

### Best fitting phylogenetic relationship of African populations

The phylogenetic relationship of all African populations was constructed under the neighbor-joining framework and TREEMIX method [27], which leverages genome-wide allele frequency data. First, we used the human ancestral alleles (ftp://ftp.1000genomes.ebi.ac.uk/vol1/ftp/technical/retired_reference/ancestral_alignments/) as outgroups in our NJ phylogenic analysis. A set of 4,587,274 SNPs, which have high-quality ancestral alleles, were randomly selected for further phylogenetic analysis. When multiple samples were sequenced in a population, we generated a consensus sequence of each population using BioEdit version 7.2.5. The consensus sequences were used as input of MEGA (version 6) [76], and the robustness of the topology was evaluated using 100 bootstrap replicates. Phylogenetic relationships and admixture across the 44 African populations were analyzed using TREEMIX [27] with the Altai Neandertal genome sequence used as an outgroup. Variants with no more than 10% missing data in the African samples were LD-pruned using Plink version 1.9 with the parameters --indep-pairwise 50 10 0.1. These data were merged with the Altai Neandertal genome [77], leaving a final set of 5,158,190 variants. TREEMIX version 1.13 was run for 0–10 migrations, rooted by the Altai Neandertal individual, and using the parameters -global -bootstrap -noss -k 500.

### Effective population size and divergence time analyses

We estimated the *Ne* and divergence time between populations using MSMC, which is a multiple sequentially Markovian coalescent method to infer effective population size and separation time between populations [38]. Since MSMC requires haplotypes as input, we phased the SNPs from the VCF files with SHAPEIT version 2.r837 [78] using the haplotypes of African populations in the 1000 Genomes Project phase 3 [20] as the reference panel (with parameters --no-mcmc, --input-ref, --include-grp AFR, --effective-size 17469, -window 0.5). We left the heterozygous sites that were not reported in the 1000 Genomes Project as unphased. This phasing strategy is the same as was used in the original SGDP study [14].

Following the instructions of MSMC, both the unphased and phased heterozygous sites were converted to the required input format [38]. We estimated the *Ne* for each sample using both phased and unphased sites. The divergence time estimation between populations was inferred with two phased genomes (one individual per population), and all the unphased sites were excluded using the “--skipAmbiguous” parameter [38]. MSMC reports the scaled population size by twice the mean autosomal per generation mutation rate *μ*, and time-scaled by the mutation rate per year ν, where *μ* = νg and *g* is the generation time. In this study, we scaled the *Ne* size by 2 *μ =* 2.5 × 10^−8^, assuming mutation rate per generation ν = 4.3 × 10^−10^ and generation time *g* = 29 years. We define that the divergence of two populations based on when the relative cross-coalescence rate drops to 0.5 as in [38].

### Scans for local adaptation

We first merged the populations into six meta-ancestry groups and then calculated a per site *F*_ST_ statistic adjusted for small sample sizes [58] between all group pairs for all SNPs with no more than 20% missing data in any group. For each remaining SNP in each group *i*, the statistic ${d}_i=\sum \limits_{j\ne i}^j{\left({F}_{\mathrm{ST}}\left(i,j\right)-E\left[{F}_{\mathrm{ST}}\left(i,j\right)\right]\right)}^2/\mathrm{sd}\left[{F}_{\mathrm{ST}}\left(i,j\right)\right]$ was calculated, where *E*[*F*_ST_(*i*, *j*)] is the mean and sd[*F*_ST_(*i*, *j*)] is the standard deviation of the *F*_*ST*_ between populations *i* and *j*. Outlier variants were defined as *d* values within the top 0.1% of the empirical distribution. To identify independent outliers (i.e., that are not in LD), percentiles were calculated for all variants (percent of variants with a *d* value higher than a given variant) and the “—clump” command from plink v1.9 was used to cluster independent groups of outliers (with parameters --clump-p1 0.001 --clump-p2 0.01 --clump-kb 1000). This returned a set of independent “tag” variants for each independent cluster. All variants in strong LD (*r*^2^ > 0.8) with these tags were considered as potential locally adaptive. Low sample size per population limits use of methods to detect signatures of natural selection based on the allele frequency spectrum or extended haplotype homozygosity [79, 80].

To test for functional enrichment of outlier variants, functional genomic regions including DNase I hypersensitive sites (DHS) annotated as promoters, enhancers, and dyadic regions [59]; enhancers identified using Cap Analysis of Gene Expression (CAGE) [60]; genic regions including exons, introns, 3′ UTRs, and 5′ UTRs [61]; and conserved regions [61] were overlapped with outlier variants using GoShifter [63]. Ten thousand permutations were performed for each genomic category, and *P* values were calculated as the number of permuted scores higher than the observed score, with the *P* values less than the 0.05 family-wise error rate (*P* < 5.56 × 10^−3^) considered significant (Bonferroni-corrected for the number of annotations tested). To identify biological functions of genes near outlier windows, regions spanning all variants in strong LD with tag variants were identified and merged. These merged windows were used as test regions in GREAT with default parameters [65]. All terms belonging to “GO Molecular Function”, “GO Biological Process,” “GO Cellular Component,” “Mouse Phenotype,” “Human Phenotype,” and “Disease Ontology” with binomial and hypergeometric FDR less than 0.05 and fold enrichment greater than 2 are presented.

## Additional files

Additional file 1: **Table S1.** Sample information in this study. (XLSX 12 kb)

Additional file 2: **Figure S1.** A phylogeny of African lineages used the Altai Neandertal as outgroup constructed using Treemix allowing for six migration events. **Figure S2.** Principal component analysis of 44 African and 32 west Eurasian populations using principal component analysis. **Figure S3.** ADMIXTURE analysis of 92 African and 62 West Eurasian individuals from *K* = 2 to 10. **Figure S4.** Effective population size of African Khoesan-speaking populations. (PDF 414 kb)

Additional file 3: **Table S2.** Enrichment test results of positively selected loci in different populations using GREAT. (XLSX 58 kb)
