# Correction to: African evolutionary history inferred from whole genome sequence data of 44 indigenous African populations

**DOI:** 10.1186/s13059-019-1821-1

**Published:** 2019-10-09

**Authors:** Shaohua Fan, Derek E. Kelly, Marcia H. Beltrame, Matthew E. B. Hansen, Swapan Mallick, Alessia Ranciaro, Jibril Hirbo, Simon Thompson, William Beggs, Thomas Nyambo, Sabah A. Omar, Dawit Wolde Meskel, Gurja Belay, Alain Froment, Nick Patterson, David Reich, Sarah A. Tishkoff

**Affiliations:** 1 Department of Genetics, University of Pennsylvania, Philadelphia, PA 19104 USA; 2 Present Address: State Key Laboratory of Genetic Engineering, Human Phenome Institute, School of Life Sciences, Fudan University, 2005 Songhu Road, Shanghai, China; 3 Department of Genetics, Harvard Medical School, Boston, MA 02115 USA; 4 Broad Institute of Harvard and MIT, Cambridge, MA 02142 USA; 5 Howard Hughes Medical Institute, Harvard Medical School, Boston, MA 02115 USA; 6 Present Address: Division of Genetic Medicine, Vanderbilt University Medical Center, Vanderbilt University, Nashville, TN 37232 USA; 7 Department of Biochemistry, Muhimbili University of Health and Allied Sciences, Dares Salaam, Tanzania; 8 Center for Biotechnology Research and Development, Kenya Medical Research Institute, Nairobi, Kenya; 9 Department of Biology, Addis Ababa University, Addis Ababa, Ethiopia; 10 UMR 208, IRD-MNHN, Musée de l’Homme, Paris, France; 11 Department of Biology, University of Pennsylvania, Philadelphia, PA 19104 USA

**Correction to: Genome Biology (2019) 20:82**

**https://doi.org/10.1186/s13059-019-1679-2**

Following publication of the original article [1], a typographical error in the formula for calculating *d*_*i*_ in the “Scans for local adaptation” subsection in the Method section, was identified. The correct formula should be

$$ {d}_i=\sum \limits_{j\ne i}\left({F}_{st}\left(i,j\right)-E\left[{F}_{st}\left(i,j\right)\right]\right)/ sd\left({F}_{st}\left(i,j\right)\right) $$

The authors apologize for any confusion this error may have caused.
