# Supplementary material for: African evolutionary history inferred from whole genome sequence data of 44 indigenous African populations
Source: Genome Biol. 2019 Apr 26;20:82. doi: 10.1186/s13059-019-1679-2 (PMC6485071; doi:10.1186/s13059-019-1679-2)
Supplement: Supplementary file 2 — Figure S1. A phylogeny of African lineages used the Altai Neandertal as outgroup constructed using Treemix allowing for six migration events. Figure S2. Principal component analysis of 44 African and 32 west Eurasian populations using principal component analysis. Figure S3. ADMIXTURE analysis of 92 African and 62 West Eurasian individuals from K = 2 to 10. Figure S4. Effective population size of African Khoesan-speaking populations. (PDF 414 kb) [file 13059_2019_1679_MOESM2_ESM.pdf]

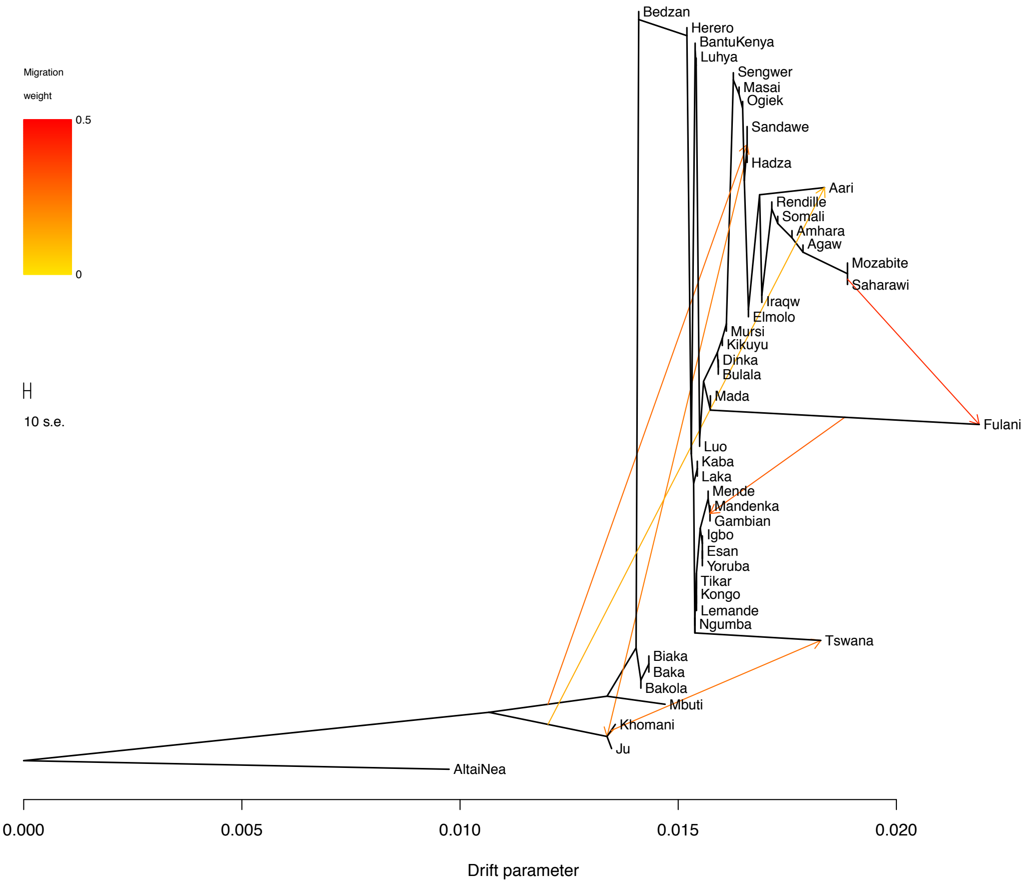

Fig S1. A phylogeny of African lineages used the Altai Neandertal as outgroup constructed using Treemix allowing for six migration events (the number of migration events for which we observed consistent results). This phylogeny largely recapitulated the phylogeny shown in Figure 2 and admixture events identified with STRUCTURE (Figure 4). For example, we observe migration between the North African Saharawi and the Fulani and between the Khomani San and the neighboring Tswana population. We also observed evidence for migration between the Hadza and the ancestors of the Khomani and Ju'huansi San and migration from a branch leading to the Western and Eastern Pygmies into the ancestors of the Hadza and Sandawe. In addition, we observe, to a lesser extent, migration from the branch leading to the San into the Aari Omotic speaking population in Ethiopia.

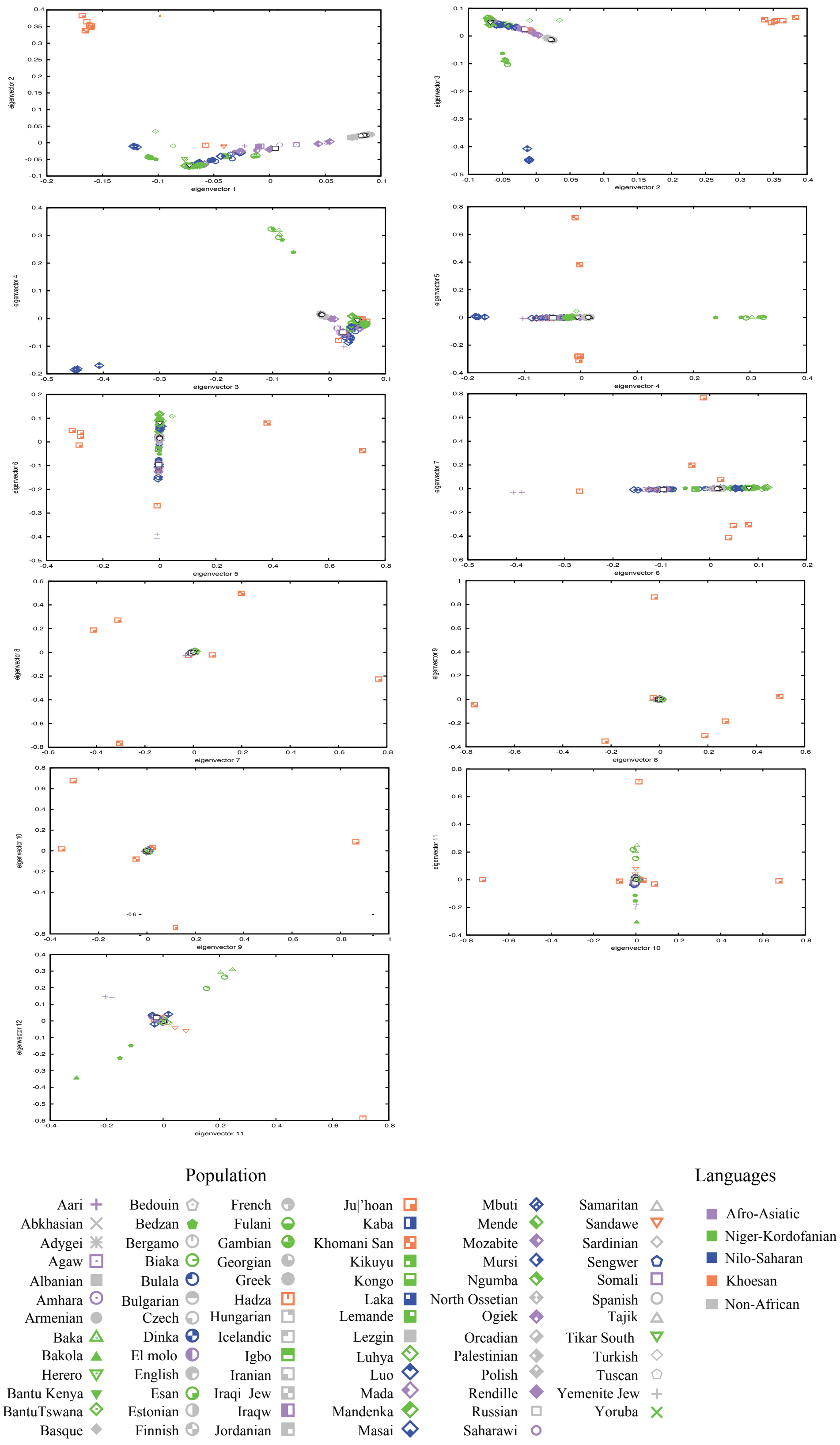

Fig S2. Principal component analysis of 44 African and 32 west Eurasian populations using principal component analysis. Each dot is an individual. The first twelve PCs are shown.

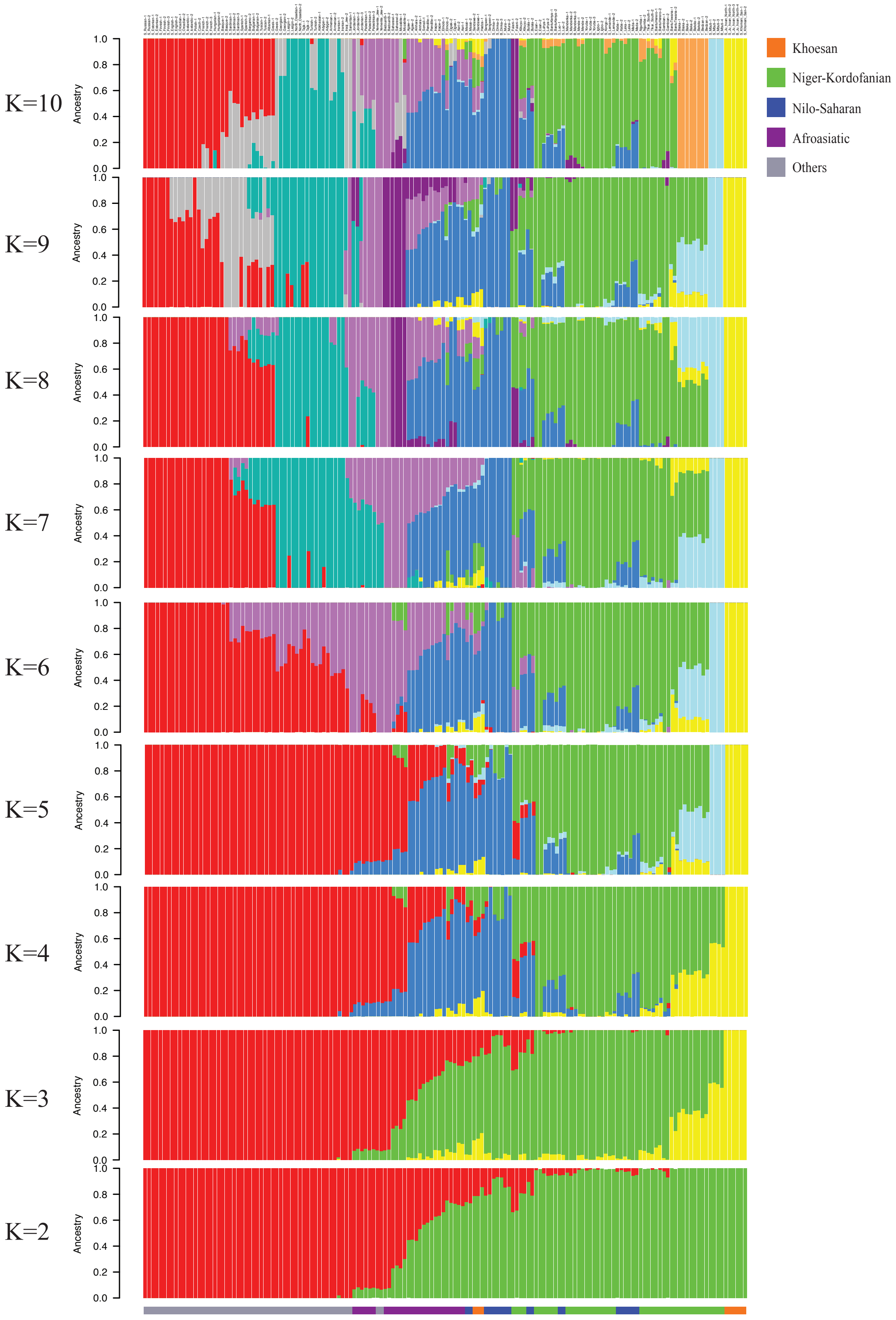

Fig S3. ADMIXTURE analysis of 92 African and 62 West Eurasian individuals from K=2 to 10. Each bar is a sample and colors represent the proportion of inferred ancestry from K ancestral populations. The lower bar represents the language classification of each sample.

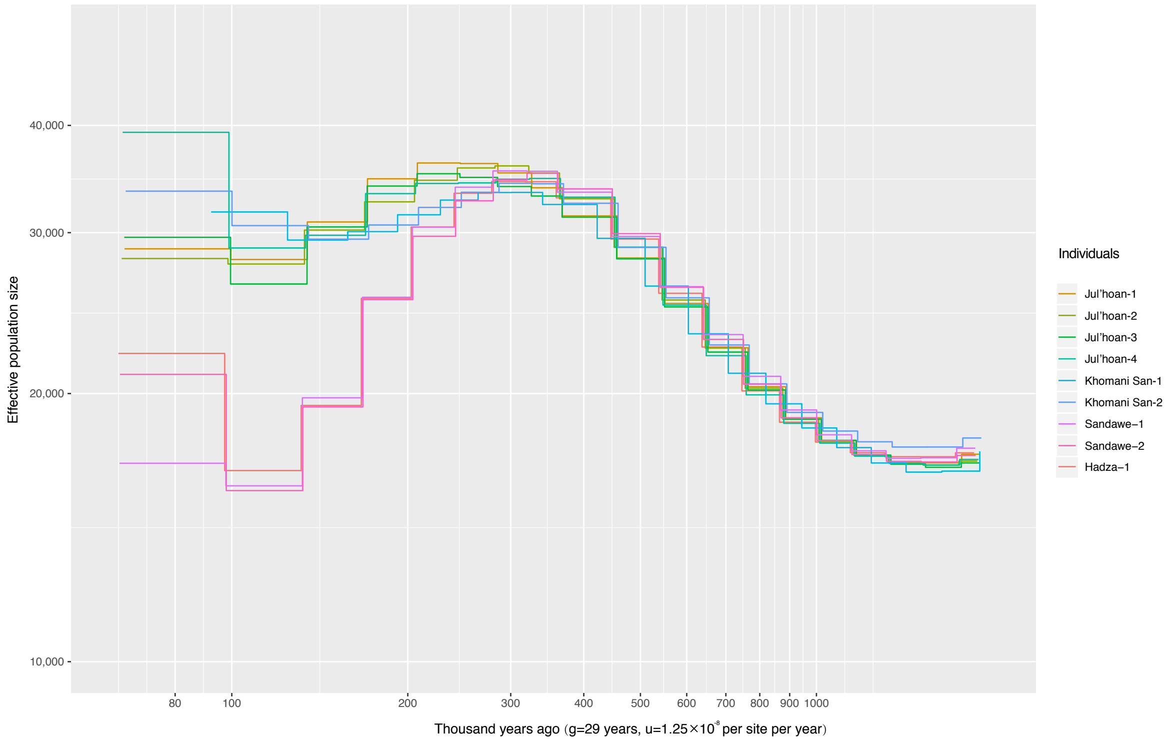

Fig S4. Effective population size of African Khoesan-speaking populations. Each line is an individual. We assume average generate time 29 years and a mutation rate per generate (v) $1.25x10^{-8}$.
